# IgA Antibodies Reveal Covert Infection With Mpox Virus in People Living With HIV in the Community: A Prospective Longitudinal Cohort Survey

**DOI:** 10.1002/mco2.70679

**Published:** 2026-03-15

**Authors:** Rui Song, Danyang Li, Lan Chen, Xiao Wang, Qiao Zhang, Zhixia Gu, Xueqi Chi, Yuanyuan Zhang, Jing Han, Li Guo, Ronghua Jin, Lili Ren, Jianwei Wang

**Affiliations:** ^1^ Beijing Ditan Hospital, Capital Medical University Beijing People's Republic of China; ^2^ NHC Key Laboratory of Systems Biology of Pathogens and Christophe Mérieux Laboratory Chinese Academy of Medical Sciences & Peking Union Medical College National Institute of Pathogen Biology Beijing People's Republic of China; ^3^ Key Laboratory of Pathogen Infection Prevention and Control (Ministry of Education) State Key Laboratory of Respiratory Health and Multimorbidity National Institute of Pathogen Biology Chinese Academy of Medical Sciences & Peking Union Medical College Beijing People's Republic of China; ^4^ Key Laboratory of Respiratory Disease Pathogenomics Chinese Academy of Medical Sciences & Peking Union Medical College Beijing People's Republic of China

**Keywords:** covert transmission, MPXV, people living with HIV, undetected infection

## Abstract

Covert mpox virus (MPXV) infection among people living with HIV (PLWH) remains poorly understood. This study aimed to investigate undetected MPXV infections through seroepidemiological analysis. We recruited 148 PLWH during July 2–9, 2023 (baseline), with 60 and 148 participants returning for 4‐ and 14‐month follow‐ups, respectively. PCR testing showed that all saliva and blood samples were negative for MPXV‐DNA. The titers of IgG, IgA, and IgM were evaluated using ELISA with virions. MPXV‐IgA and IgM were undetectable, 15 participants born before 1980 had low MPXV‐IgG at baseline. At 14 months, 10 participants (6.8%) showed increased MPXV‐IgG titers. All seroconverters had detectable neutralizing antibodies, and nine were MPXV‐IgA positive. Further results showed that IgG seropositivity against MPXV proteins (A29L, A35R, B6R, E8L, H3L, and M1R) ranged from 0% to 80%, while IgA seropositivity ranged from 0% to 60% among the 10 participants at the 14‐month. The B6R and E8L combination showed IgG detection comparable to whole virions, while E8L and H3L combination increased IgA seropositivity to 70%. The occurrence of MPXV covert infection among PLWH underscores the need to improve surveillance strategies in the community. The presence of MPXV‐IgA in blood may offer a preliminary temporal signal of MPXV infection.

## Graphical abstract

This study identified covert infection of MPXV among people living with HIV and MPXV‐IgA was the effective indicator. The combined use of two antigen protein set of MPXV (B6R plus E8L and E8L plus H3L) yield IgA test results comparable to those obtained with the MPXV. These findings provide surveillance strategies to evaluate MPXV transmission in the high‐risk populations.

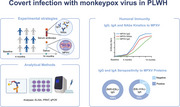

## 1 Introduction

Since May 2022, Mpox has spread globally and is transmitted primarily from person to person through close contact with individuals infected with mpox virus (MPXV). The virus predominantly affects men who have sex with men (MSM), and PLWH are a high‐risk population after exposure to MPXV. It has been estimated that more than 50% of mpox cases occur in PLWH [1, 2, 3].

Mpox typically presents with mucocutaneous lesions, fever, swollen lymph nodes, and myalgia [4]. Some patients infected with MPXV are asymptomatic or have mild infections with minimal signs and symptoms [5]. A study from San Francisco, California, indicated that there were three possible undetected MPXV infections among 209 participants, as determined by the assessment of antiorthopoxvirus IgG and IgM [6]. A study conducted in Rome revealed that 21 participants positive for MPXV‐IgG among 285 PLWH had no reported MPXV infection, history of vaccination, or clinical manifestations associated with mpox [7]. After retrospective screening of samples from men attending a sexual health clinic in Belgium, 4 of 224 anorectal swabs were positive for MPXV‐DNA; three of these individuals reported no symptoms, but three men had confirmed MPXV exposure according to serological analysis [8]. Thus, a proportion of mpox cases remain undetected due to atypical symptoms, relatively mild illness, and an unwillingness to seek hospital care.

The MPXV comprises two main clades: Clade I (Central African/Congo Basin strain) and Clade II (West African strain) [9]. Clade IIb primarily affects MSM, whereas Clade Ib exhibits distinct transmission dynamics, with infections showing a more balanced sex distribution across populations [10]. Mpox was announced as a public health emergency of international concern twice, on July 23, 2022 [11] and August 14, 2024 [12], prompting investigations of its spread in the community to improve disease control strategies. However, the available data are based mainly on cross‐sectional studies conducted in a short window after viral exposure via testing for IgM and DNA. Investigations of undetected infections in populations with a history of smallpox vaccination are urgently needed. Moreover, Current evaluations of MPXV‐specific antibodies have primarily focused on the acute‐phase antibody response in individuals with mpox [13, 14]. Moraes‐Cardoso et al. assessed the durability of IgG and IgA titers against MPXV proteins (35R, H3L, and A29L) over a period of 182 days [15].

Here, we designed a prospective longitudinal study and recruited PLWH from the Red Ribbon Center. The presence of IgG, IgA, IgM, and neutralizing antibodies against MPXV was evaluated using inactivated virions and antigen proteins. We aimed to identify host indicators to detect covert MPXV infections in high‐risk populations in the community in China. The findings from our prospective longitudinal study indicate that PLWH are at high risk of MPXV infection. The high rate of asymptomatic or mild cases following MPXV exposure among PLWH supports the occurrence of covert MPXV infection in this population. These results highlight the importance of strengthening surveillance strategies for high‐risk groups through seroepidemiological analyses to improve the control of MPXV transmission.

## 2 Results

### 2.1 Participants and Samples

**FIGURE 1:**
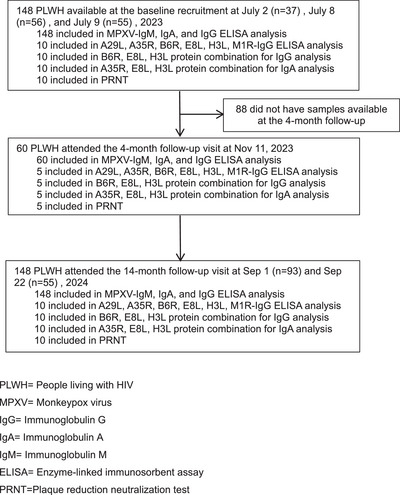
Study flow chart.

A total of 148 PWLH were recruited and followed up at 14 months, and 60 of them were also followed up at 4 months (Figure 1). The median age of the participants was 45 years (IQR: 36–53), and 124 (83.8%) were male (Table 1). Seventy‐nine (53.4%) of them were born before 1980. Only 34 (23.0%) had undetectable HIV loads. Ten participants had low CD4 cell counts (< 350 cells/mm^3^), whereas 21 (14.2%) had 350–499 CD4 cells/mm^3^. Sexually transmitted diseases were found in 19 (12.8%) participants (Table 1). None of these participants had MPXV infection at baseline. Plasma and saliva samples were collected from each participant at baseline and at the 14‐month follow‐up, and from 60 (40.5%) participants at the 4‐month follow‐up.

**TABLE 1:** Demographic and clinical characteristics of the participants.

Characteristic	Total (*n* = 148)	MPXV‐IgG (−) (*n* = 138)	MPXV‐IgG (+) (*n* = 10)	*p* value
Age (year)				
Median (IQR)	45 (36–53)	45 (37–53)	41 (35–48)	0.57
Sex, *n* (%)				
Male	124 (83.8%)	114 (82.6%)	10 (100%)	0.37
Female	24 (16.2%)	24 (17.4%)	0 (0%)	
Years from HIV diagnosis, *n* (%)				
1–3	25 (16.9)	23 (16.7%)	2 (20%)	0.68
> 3	123 (83.1%)	115 (83.3%)	8 (80%)	
CD4 cell count (cells/mm^3^), *n* (%)				
< 350	10 (6.8%)	10 (7.2%)	0 (0%)	> 0.99
350–499	21 (14.2%)	21 (15.2%)	0 (0%)	0.36
≥ 500	117(79.1%)	107 (77.5)	10 (100%)	0.12
HIV viral load strata (RNA copies per mL), *n* (%)				
Undetectable	34 (23%)	32 (23.2%)	2 (20%)	0.67
Detected but ≤ 1000	b24 (16.2%)	22 (15.9%)	2 (20%)	0.67
> 1000	90 (60.8%)	84 (60.9%)	6 (60%)	> 0.99
Concurrent STDs, *n* (%)				
Syphilis	17 (11.5%)	15 (10.9%)	2 (20%)	0.32
Gonorrhea	1 (0.7%)	1 (0.7%)	0 (0%)	> 0.99
*Condyloma acuminatum*	2 (1.4%)	2 (1.4%)	0 (0%)	> 0.99
Genital herpes	0 (0%)	0	0 (0%)	> 0.99
Other STDs	0 (0%)	0	0 (0%)	> 0.99
None of the above	129 (87.2%)	121 (87.7%)	8 (80%)	0.62

Abbreviation: STDs, sexually transmitted diseases.

### 2.2 Temporal Changes in Antibody Titers Against MPXV Indicate Covert MPXV Infection in PLWH

**FIGURE 2:**
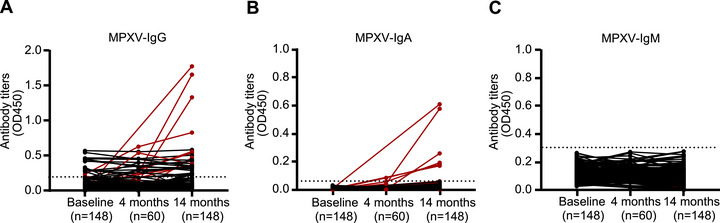
Kinetics of IgG, IgA, and IgM antibody titers against MPXV in people living with HIV. The kinetics of MPXV‐ IgG (A), IgA (B), and IgM (C) titers were assessed in 148 participants from baseline to 14 months post‐enrollment. Antibody titers were determined using enzyme‐linked immunosorbent assay (ELISA), with lysate from MPXV‐infected cells serving as the coating antigen.

To determine whether the participants were exposed to MPXV during the study period, we first tested for MPXV‐DNA in saliva and blood samples collected at baseline and throughout the follow‐up period. All the samples were negative for MPXV‐DNA (data not shown). We further analyzed IgG, IgM, and IgA levels in the plasma using lysed MPXV cultured in Vero cells, as well as NAb levels using cultured MPXV. Fifteen participants had low levels of IgG against MPXV at baseline (Figure 2A). All these individuals were born before 1980 and reported a history of smallpox vaccination. All the plasma samples were negative for MPXV‐IgA (Figure 2B) and IgM (Figure 2C) at baseline, which further confirmed that the participants were not infected with MPXV at baseline.

**FIGURE 3:**
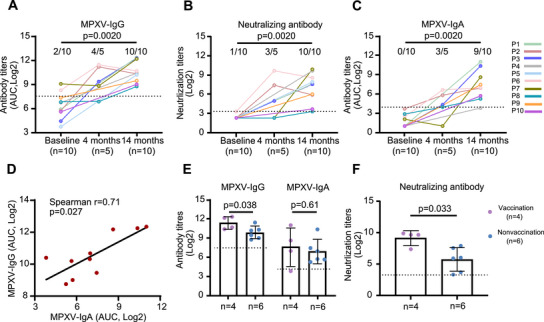
Kinetics of IgG, IgA, and neutralizing antibody titers against MPXV in 10 participants. (A–C) Kinetics of MPXV‐IgG (A), neutralizing antibody (B), and MPXV‐IgA (C) titers in 10 participants. (D) Correlation between MPXV‐IgA and MPXV‐IgG titers. (E–F) MPXV‐IgG, MPXV‐IgA (E), and neutralizing antibody (F) titers in participants with or without a history of smallpox vaccination. MPXV‐IgG and IgA titers were determined using ELISA, with lysate from MPXV‐infected cells serving as the coating antigen. Neutralizing antibody titers were assessed using the PRNT. The Wilcoxon matched‐pairs signed rank test was used to compare antibody titers at baseline and follow‐up. Comparisons of antibody titers between participants born before and after 1980 were conducted using the Mann–Whitney *U* test. Spearman correlation analysis was used for assessing correlations. The dashed lines represent the assay detection limits.

After 14 months, 10 (6.8%) of the participants (referred to as P1 through P10) had significantly greater plasma MPXV‐IgG titers than they did at baseline (median OD450: 0.55 vs. 0.027, *p* = 0.0020, Figure 2A; median AUC: 1315 [IQR: 655.6–4701] vs. 83.1 [41.6–199.7], *p* = 0.0020, Figure 3A). All 10 participants also had detectable NAbs at 14 months compared with baseline (median titers: 216.7 [IQR 48.2–484.5] vs. negative, *p* = 0.0020; Figure 3B). Compared with the levels observed at baseline, 9 of the 10 participants with seroconversion presented increasing levels of MPXV‐IgA (median AUC 124.9 [IQR 46.9–627.2] vs. 1 [1.0–5.9], *p* = 0.0020; Figure 3C). Furthermore, MPXV‐IgA titers were significantly correlated with MPXV‐IgG titers (Spearman *r* = 0.71, *p* = 0.027; Figure 3D). Among the 10 participants, 4 had a history of smallpox vaccination and exhibited higher levels of MPXV‐IgG (3137 [1424–5067] vs. 937.9 [493.1–1656], *p* = 0.038) and NAb (485.9 [152.2–1551] vs. 43.7 [9–212.6], *p* = 0.033) titers compared to those without smallpox vaccination history (Figure 3E). However, no significant difference was observed in MPXV‐IgA titers between the two groups (*p* = 0.61; Figure 3F). In addition, MPXV‐IgM levels in each 10 participants showed no significant difference across baseline and follow‐ups (Figure S1).

Five of the 10 participants (P2, P3, P6, P7, and P8) were also followed up at 4 months. Compared with baseline, we detected seroconversion for IgG (2323 and 651.4, respectively) and NAbs (169 and 31.2, respectively) in two participants (P2 and P3) (Figure 3A,B) and seroconversion for IgA in three participants (P2, P3, P6) (56.3, 20, 99.7, respectively; Figure 3C). The titers of MPXV‐IgG and NAbs in P6 participants, who were positive for IgG and NAbs at baseline, increased significantly at 4 months (310.6 vs. 2781 for MPXV‐IgG; 10 vs. 810 for NAb; Figure 3A,B). These results suggest that three participants (P2, P3, and P6) were exposed to MPXV prior to the 4‐month follow‐up.

Among the three participants (P2, P3, and P6) who were exposed to MPXV before the 4‐month follow‐up, titers of MPXV‐IgG decreased in P2 (2323 vs. 1277) and P6 (2781 vs. 1635) (Figure 3A), as did NAbs (P2: 169 vs. 13.3; P6: 810 vs. 376; Figure 3B). However, the IgA titers remained stable between 10‐month follow‐up (P2: 56.3 vs. 123; P6: 99.7 vs. 126.1; Figure 3C). P3 presented increasing titers of MPXV‐IgG (AUC, 651.4 vs. 2794; Figure 3A), and NAbs (GMT, 31.3 vs. 264.2; Figure 3B), and MPXV‐IgA (AUC, 20.1 vs. 1325; Figure 3C) at the 14‐month follow‐up. These results indicate that IgA may be an effective host indicator for evaluating recent MPXV infections, regardless of smallpox vaccination history.

### 2.3 Clinical Symptoms of Covert MPXV Infection in PLWH

To evaluate MPXV infection status, we further reviewed the demographic information and clinical symptoms of the patients. The median age of the 10 participants was 41 years (IQR 35–48), and all were male. The median CD4^+^ T‐cell count was 815 cells/mm^3^ (range, 611–1026). There were no statistically significant differences in age (*p* = 0.57), sex (*p* = 0.37), years from HIV diagnosis (*p* = 0.68), CD4 cell count (< 350 cells/mm^3^: *p *> 0.99; 350–499 cells/mm^3^: *p* = 0.36; ≥ 350 cells/mm^3^: *p* = 0.12, respectively), and viral load of HIV (undetectable: *p* = 0.67; detected but ≤1000: *p* = 0.67; >1000: *p *> 0.99, respectively) between MPXV‐IgG‐positive and MPXV‐IgG‐negative participants (Table 1). Four of (P1, P4, P6, and P7) 10 participants were born between 1969 and 1980 (Table 1). Four participants self‐reported suffering mild symptoms, including diarrhea (4), fever (2), and skin lesions (1). Only one participant asked for medical care (Table S2). None of them or their partners were diagnosed with mpox. These findings suggest the occurrence of covert MPXV infection in PLWH in the community.

### 2.4 Antibody Responses Against Proteins of MPXV in PLWH

**FIGURE 4:**
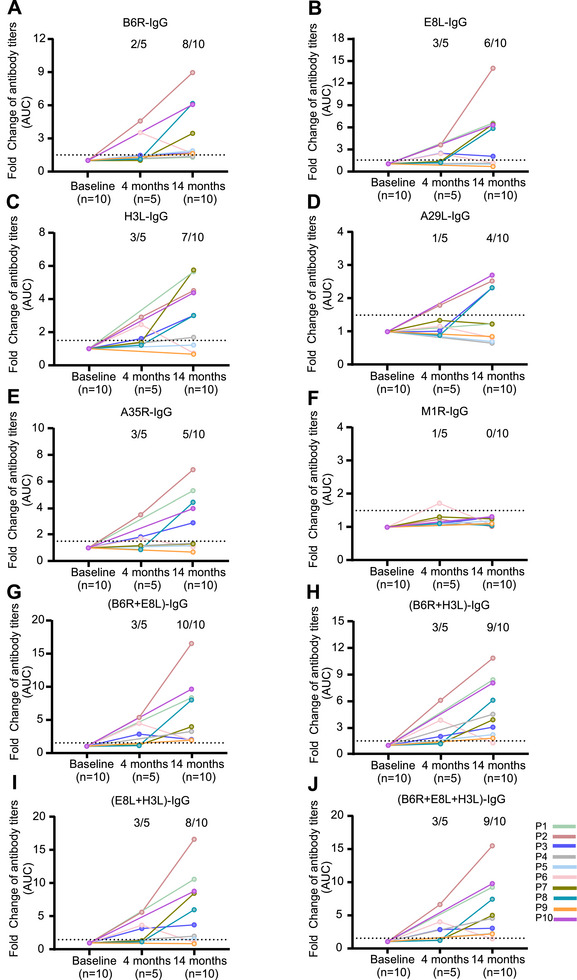
Kinetics of IgG titers against MPXV proteins in people living with HIV. The kinetics of B6R‐IgG (A), E8L‐IgG (B), H3L‐IgG (C), A29L‐IgG(D), A35R‐IgG(E), M1R‐IgG(F), (B6R+E8L)‐IgG (G), (B6R+H3L)‐IgG (H), (E8L+H3L)‐IgG (I), and (B6+E8+H3)‐IgG (J) titers in 10 participants. The IgG antibody titers were determined using ELISA, with various MPXV proteins or protein combinations serving as the coating antigens. The dotted lines indicate a fold change ≥ 1.5.

**FIGURE 5:**
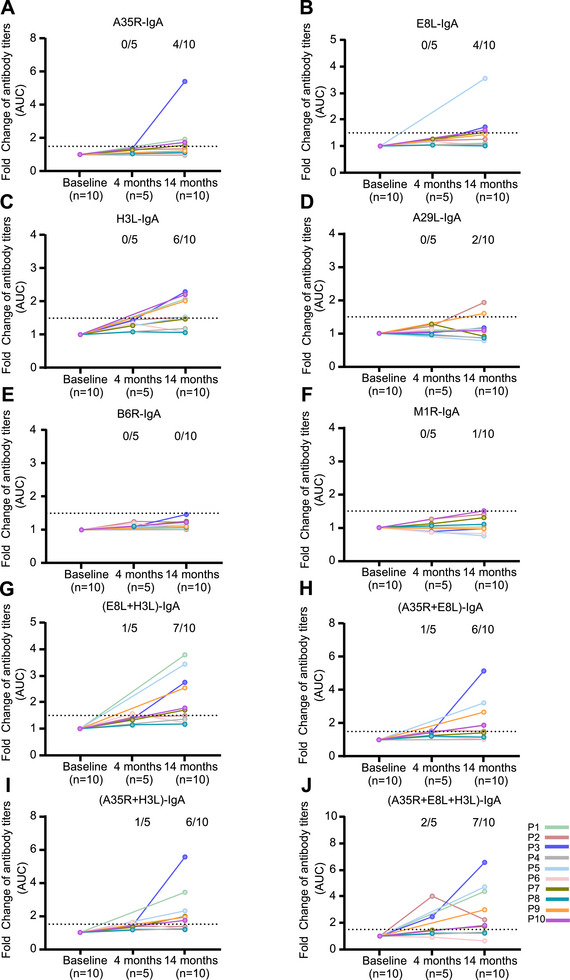
Kinetics of IgA titers against MPXV proteins in people living with HIV. The kinetics of A35R‐IgA (A), E8L‐IgA (B), H3L‐IgA (C), A29L‐IgA (D), B6R‐IgA (E), M1R‐IgA (F), (E8L+H3L)‐IgA (G), (A35R+E8L)‐IgA (H), (A35R+H3L)‐IgA (I), and (A35R+E8L+H3L)‐IgA (J) titers in 10 participants. The IgA antibody titers were determined using ELISA, with various MPXV proteins or protein combinations serving as the coating antigens. The dotted lines indicate a fold change ≥ 1.5.

**FIGURE 6:**
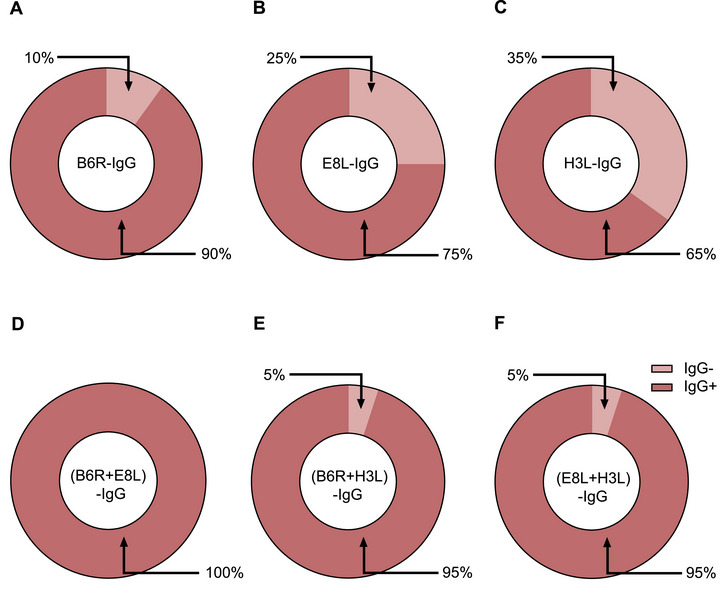
Positivity rate for IgG against different MPXV proteins in patients with mpox. Positivity rates of B6R‐IgG (A), E8L‐IgG (B), H3L‐IgG (C), (B6R+E8L)‐IgG (D), (B6R+H3L)‐IgG (E), and (E8L+H3L)‐IgG (F) in 20 patients with mpox. The IgG antibody titers were determined using ELISA, with various MPXV proteins or protein combinations serving as the coating antigens.

**FIGURE 7:**
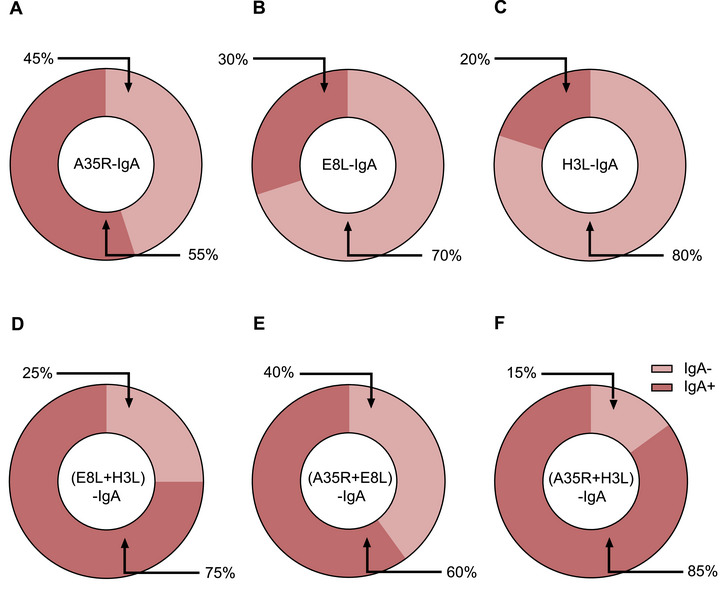
Positivity rate for IgA against different MPXV proteins in patients with mpox. Positivity rates of A35R‐IgA (A), E8L‐IgA (B), H3L‐IgA (C), (E8L+H3L)‐IgA (D), (A35R+E8L)‐IgA (E), and (A35R+H3L)‐IgA (F) in 20 patients with mpox. The IgA antibody titers were determined using ELISA, with various MPXV proteins or protein combinations serving as the coating antigens.

To determine whether the efficacy of MPXV antigen proteins was similar to that of virions in terms of evaluating humoral responses, we analyzed the antibody titers in plasma samples and six MPXV proteins. Among the 10 plasma samples, 8 (80%), 6 (60%), 7 (70%), 4 (40%), 5 (50%), and 0 (0%) exhibited significant increases in IgG titers against B6R, E8L, H3L, A29L, A35R, and M1R, respectively, at 14 months compared with those at baseline (Figure 4A–F). The antigen proteins were then combined to test their efficacy. The results showed that the combination of the B6R and E8L proteins achieved efficacies comparable to those of virions when tested with IgG (Figure 4G), whereas IgG specific for the combination of the B6R and H3L proteins, E8L and H3L, and B6R plus E8L and H3L proteins were detectable in 9/10 (90%) (Figure 4H), 8/10 (80%) (Figure 4I), and 9/10 (90%) (Figure 4J) plasma samples, respectively. Corresponding increases in IgA titers against A35R, E8L, H3L, A29L, B6R, and M1R were observed in four (40%) (Figure 5A), four (40%) (Figure 5B), six (60%) (Figure 5C), two (20%) (Figure 5D), zero (0%) (Figure 5E), and one (10%) (Figure 5F) plasma samples, respectively. The combination of E8L and H3L increased the detection rate of IgA to 70% (Figure 5G), whereas IgA specific for the combination of the A35R and E8L proteins, A35R and H3L, and A35R plus E8L and H3L proteins were detectable in 6/10 (60%) (Figure 5H), 6/10 (60%) (Figure 5I), and 7/10 (70%) (Figure 5J) plasma samples, respectively. The efficacy of the combined antigen proteins in detecting IgG against B6R+H3L, B6R+ H3L, and E8L+H3L, as well as IgA against E8L+H3L, A35R+E8L, and A35R+H3L, was further validated using 20 plasma samples obtained from mpox patients. The results showed that the use of combined antigen proteins enhanced the detection rates of both IgG (Figure 6A–F) and IgA (Figure 7A–F) antibodies against MPXV.

## 3 Discussion

We performed a prospective longitudinal study to evaluate MPXV infections in PLWH in the community. All the participants had no history of MPXV infection according to MPXV‐DNA and antibody testing at baseline. After 14 months, on the basis of increasing titers of IgA, IgG, and NAbs, we determined that 6.8% (10/148) of the participants had experienced recent MPXV infection. All PLWH with seroconversion were asymptomatic or had mild symptoms, and their partners had no reported symptoms. MPXV‐IgA would be an effective indicator for the surveillance of recent MPXV infections within the past 10 months, especially in populations with a history of smallpox vaccination. The combination of the antigen proteins B6R and E8L or E8L and H3L could achieve efficacies comparable to those of MPXV virions when evaluating IgG and IgA, respectively.

It is unclear whether asymptomatic MPXV‐infected patients are contagious. The transmissibility of asymptomatic orthopoxvirus infection is extremely low, according to a previous study [16]. However, MPXV has been successfully isolated from asymptomatic patients, suggesting the possibility that asymptomatic individuals are contagious [8, 17]. The WHO has announced mpox as a PHEIC two times to raise concern on a global scale. Better understanding the epidemic of MPXV in the community is important for improving disease control strategies [18, 19]. As the epidemic of MPXV is at very low level in China, an effective method to detect covert infections in the community is needed, particularly in high‐risk populations with a history of smallpox vaccination [18, 20].

In this study, we observed that all individuals who tested positive for MPXV‐IgG, IgA, and neutralizing antibodies had CD4^+^ T cell counts exceeding 500 cells/mm^3^. This finding indicates a possible association between covert MPXV infection and maintained immunological competence within the studied cohort; however, further study is needed to validate this relationship. Notably, previous studies have shown that early humoral immune responses, particularly the concentration and breadth of IgG and IgA antibodies, are significantly associated with milder disease manifestations and more rapid viral clearance [15]. This may explain the absence of transmission among these individuals, although earlier reports have also indicated that infectious virus can be isolated from individuals with asymptomatic or unrecognized mpox symptoms [21].

Retrospective surveillance of MPXV infection via viral DNA testing has been reported to achieve detection of asymptomatic mpox in MSM [22, 23, 24]. However, given that the persistence of nucleic acids is very short, it is difficult to identify patients when they are asymptomatic or suffer mild symptoms. Antibody detection is particularly effective in ruling out infections and supporting clinical diagnosis [25]. Measuring the antibody titers of IgM and IgG has been the most practical method for mpox diagnosis in previous studies [6, 7, 8, 26, 27, 28]. However, the period during which IgM can be detected is approximately 5–56 days after rash onset [28], which might not be suitable for evaluating recent infections when patients have no skin lesions or when the level of MPXV infection is low. In our study, half of the participants with undetected MPXV infection participants were followed up at 4 months, but all of them were IgM negative. Moreover, the orthopoxvirus ELISA could not distinguish MPXV infection from smallpox vaccination on the basis of cross‐sectional IgG tests. We found that IgA antibodies persisted for at least 10 months on the basis of the five available plasma samples collected during follow‐up. IgA antibodies should offer a preliminary temporal signal of MPXV infection, regardless of clinical manifestations. However, the prolonged follow‐up intervals between testing sessions may have resulted in missing the optimal window for the detection of viral and specific antibodies. Furthermore, infections presenting with very mild symptoms may have been overlooked or not reported. This potential underdetection could have influenced the detection rate of MPXV covert infections.

In our study, antibodies against MPXV were analyzed using lysed virions. However, a biosafety level 3 laboratory is needed to culture the virus. The A29L, E8L, H3L, and M1R proteins of MPXV are surface proteins of the intracellular mature virion (IMV), and A35R and B6R are surface proteins of the extracellular enveloped virion (EEV). These proteins have been utilized in mpox vaccine design as protective antigens [29]. We tested the efficacy of these proteins for antibody detection. However, not all MPXV‐IgG‐ or IgA‐positive plasma samples yielded positive results when individual proteins were used, which suggests that a single protein may lead to false negative results. The underlying mechanisms responsible for the observed differences in antibody production are unclear. In this study, the combination of the B6R and E8L proteins had a positivity rate comparable to that of MPXV virions in terms of IgG responses, whereas the combination of the E8L and H3L proteins significantly increased the detection rate for IgA. Therefore, different combinations of MPXV proteins can serve as alternatives for detecting antibodies against MPXV when virions are unavailable.

Our study has several limitations. First, the sample size was relatively small and limited to a single center. The potential presence of undetected MPXV infections warrants further investigation through larger, multicenter studies. Second, the correlations of the symptoms recorded in the retrospective questionnaire for the four participants with MPXV infection need to be clarified. Third, the detection of covert MPXV infections among PLWH relied on serological testing in the absence of viral DNA confirmation, raising concerns about potential cross‐reactivity with antibodies against other orthopoxviruses. Fourth, behavioral exposure data were not available for the seroconverters.

In summary, we evaluated covert infections among PLWH via a prospective longitudinal study. The observed undetected covert infections with MPXV among PLWH underscore the need for surveillance and prevention measures. MPXV‐IgA could be used as an effective host factor for the surveillance of MPXV infections.

## 4 Materials and Methods

### 4.1 Study Design and Participants

We recruited PLWH from the Red Ribbon Centre of Beijing Ditan Hospital, Capital Medical University, Beijing, China, between July 2 and 9, 2023. Participants older than 18 years with confirmed HIV infection were eligible. The participants were followed up on November 11, 2023 (4 months), and between September 1 and 22, 2024 (14 months). All patients were subjected to HIV‐RNA and CD4 cell count testing at baseline and follow‐up. At baseline, as well as at the 4‐month and 14‐month follow‐ups, PLWH were interviewed with questionnaires on demographic and clinical characteristics. Participants who tested positive for MPXV antibodies at 14 months were asked to complete a retrospective survey questionnaire.

Saliva and venous blood were collected from each participant by clinicians at Beijing Ditan Hospital, Capital Medical University. Nucleic acids were extracted from saliva and blood samples, and MPXV DNA analysis was performed via real‐time PCR [14]. A total of 20 plasma samples from patients with mpox that were collected between June 2 and September 23, 2023, were included in this study as positive controls. All 20 samples tested positive for both MPXV‐IgA and MPXV‐IgG antibodies [14]. Six healthy donors born after 1990 were included as negative controls.

### 4.2 Plasma Isolation

Venous blood specimens were collected from PLWH and processed within 6 h for plasma isolation. Plasma separation was performed via centrifugation at 300 × *g* for 10 min, followed by storage at −80°C until subsequent analysis.

### 4.3 Enzyme‐Linked Immunosorbent Assay

MPXV‐specific IgM, IgA, and IgG in plasma were tested using ELISA with inactivated virus as previously described [14]. All commercial reagents employed in this study are detailed in Table S1. Briefly, MPXV‐infected Vero cells were lysed using RIPA buffer (Solarbio, Beijing, China), with protein concentrations measured by Pierce BCA Protein Assay Kit (Thermo Fisher Scientific, USA). Lysate aliquots containing 0.5 µg total protein were coated onto high‐binding 384‐well plates (Corning, NY, USA) using carbonate coating buffer (pH 9.6). After overnight incubation at 4°C, plates were washed for three times using PBST (0.05% Tween‐20 in PBS) before blocking with 2% BSA/PBS for 2 h at 37°C. The plasma was diluted 1:100 in 0.2% BSA/PBS and incubated for 1 h at 37°C. Horseradish peroxidase‐conjugated goat anti‐human IgM (Fc5μ‐specific; Jackson ImmunoResearch, West Grove, PA, USA), goat anti‐human IgA (α‐chain‐specific; Sigma‐Aldrich, St. Louis, MO, USA), and goat anti‐human IgG (Fc‐specific; Sigma‐Aldrich) were used as secondary antibodies at 1:60,000 dilution. Uninfected Vero cell lysates were used as a negative control for each plasma sample. Background signals from uninfected cell lysates were subtracted for MPXV specificity responses. ELISA cutoffs were established as mean +3SD of negative controls (individuals born post‐1990), corresponding to thresholds of 0.3 for MPXV‐IgM, 0.03 for MPXV‐IgA, and 0.2 for MPXV‐IgG. The area under the curve (AUC) was calculated using GraphPad Prism (Version 10.3).

IgM, IgA, and IgG targeting the MPXV proteins A29L, A35R, H3L, E8L, B6R, and M1R were assessed using ELISA. Briefly, a 25 ng/well concentration of the A29L, A35R, H3L, or E8L protein (ACROBiosystems, Beijing, China), the B6R or M1R protein (Sino Biological, Beijing, China), or different protein combinations was coated in a high‐binding 384‐well plate (Corning), and the plates were incubated overnight at 4°C. After washed with PBST, the plates were incubated with blocking buffer. The plasma samples were first diluted 1/100 and then subjected to six 2‐fold serial dilutions with 0.2% BSA, followed by incubation for 1 h at 37°C. Following washing, horseradish peroxidase‐conjugated goat anti‐human Fc5μ fragment‐specific polyclonal IgM (Jackson ImmunoResearch, West Grove, PA, USA), goat anti‐human α chain‐specific polyclonal IgA (Sigma‐Aldrich), and goat anti‐human Fc‐specific IgG antibodies (Sigma‐Aldrich) were added to the plates. After 1 h of incubation at 37°C, the plates were washed and developed with 50 µL of TMB two‐component substrate solution (Solarbio) in each well. The reaction was stopped by adding 25 µL of stop buffer (Solarbio). The OD450 was determined with EnSight. The AUC was calculated using GraphPad Prism (Version 10.3).

### 4.4 Plaque Reduction Neutralization Test

Neutralizing antibody (NAb) titers were determined through plaque reduction neutralization testing (PRNT) as described in a previous study [14]. MPXV IIb isolate was obtained from skin lesions of a mpox patient, and viral isolation and culture were conducted under biosafety Level 3 laboratory. The MPXV Clade IIb isolate IPBCAMS‐DT‐01‐2023 is representative of the currently circulating strains in China, as confirmed by genomic sequence analysis (https://ngdc.cncb.ac.cn/genbase/, accession number C_AA121816.1). Viral titers were determined using Vero cells (ATCC, CCL‐81). Plasma specimens underwent serial two‐fold dilutions (initial dilution 1:4) were pre‐incubated with 75 plaque‐forming units (PFU) of MPXV. Virus‐plasma mixtures were incubated for 1 h at 37°C before application to confluent Vero cell monolayers in 12‐well culture plates (Costar, Corning, NY, USA). Following 2‐h adsorption, inocula were replaced with 1 mL methylcellulose‐overlaid maintenance medium (DMEM supplemented with 2% FBS). Post‐72‐h incubation, viral plaques were visualized by crystal violet staining and enumerated using the Countstar Castor X1 automated counter (ALIT Life Science, Shanghai, China). The PRNT50 endpoint titer corresponded to the maximum plasma dilution achieving ≥ 50% plaque reduction relative to virus controls. Seropositivity threshold was established at 1:10 dilution. Convalescent‐phase plasma samples obtained from confirmed mpox cases positive for the MPXV IIb strain were used as positive controls, while sera collected from individuals born after 1990 served as negative controls.

### 4.5 Statistical Analysis

The demographic and clinical characteristics of the participants are presented as median with IQR for continuous variables and absolute values with percentages for categorical variables. The Wilcoxon matched‐pairs signed rank test was used to compare the antibody titers at baseline and follow‐ups. Comparisons of NAb titers between PLWH born before and after 1980 were performed the Mann–Whitney *U* test. Spearman correlation analysis was performed for correlation analyses. A two‐sided *p* value less than 0.05 was considered significant. All the statistical analyses were conducted using GraphPad Prism 10.3.

## Author Contributions

J.W., L.R., R.J., and L.G. conceived and designed the study and took responsibility for data integrity and accuracy of the data analysis. L.G., L.R., and Q.Z. did the literature review. D.L., L.G., L.C., X.W., Q.Z., and X.C. performed experiments. L.G., L.R., R.S., and D.L. did the analysis. L.G. and L.R. drafted the paper. R.S., L.G., D.L., X.W., Q.Z., Z.G., X.C., and Y.Z. completed the follow‐up work. R.S., Z.G., Y.Z., and J.H. collected the data of demographics and questionnaires. L.R., L.G., and R.S. verified the underlying data in the study. All authors read the manuscript. All authors approved the final version, had full access to all the data, and had final responsibility for the decision to submit for publication. We thank all the study participants and their families.

## Ethics Statement

The study was approved by the Institutional Review Boards of Beijing Ditan Hospital Capital Medical University (2023‐025). All experimental activities related to MPXV were conducted in a biosafety level 3 laboratory, and all procedures involving MPXV have been approved by the National Health Commission of the People's Republic of China (Approval No. 2025–56).

## Consent

Each participant provided written informed consent.

## Conflicts of Interest

The authors declare no conflicts of interest.

## Supporting information




**Figure S1**: Kinetics of MPX‐ IgM titers in 10 participants.
**Table S1**: Reagents used in this study.
**Table S2**: Demographics, HIV status and self‐reported symptoms of the 10 PLWH with seroconversion.

## Data Availability

The data of individual deidentified participants will not be shared but are available on request to the corresponding authors.
