# Supplementary material for: IgA Antibodies Reveal Covert Infection With Mpox Virus in People Living With HIV in the Community: A Prospective Longitudinal Cohort Survey
Source: MedComm (2020). 2026 Mar 15;7(4):e70679. doi: 10.1002/mco2.70679 (PMC13042417; doi:10.1002/mco2.70679)
Supplement: Supplementary file 1 — Figure S1: Kinetics of MPX‐ IgM titers in 10 participants. Table S1: Reagents used in this study. Table S2: Demographics, HIV status and self‐reported symptoms of the 10 PLWH with seroconversion. [file MCO2-7-e70679-s001.docx]

**Supplementary Appendix**

**IgA antibodies reveal covert infection with mpox virus in people living with HIV in the community: a prospective longitudinal cohort survey**

Rui Song^1,#^ | Danyang Li^2,#^ | Lan Chen^2^ | Xiao Wang^2^ | Qiao Zhang^2^ | Zhixia Gu^1^ | Xueqi Chi^2^ | Yuanyuan Zhang^1^ | Jing Han^1^ | Li Guo^2,3,4,*^ | Ronghua Jin^1,*^ | Lili Ren^2,3,4*^ | Jianwei Wang^2,4*^

^1^Beijing Ditan Hospital Capital Medical University, Beijing, P. R. China.

^2^NHC Key Laboratory of Systems Biology of Pathogens and Christophe Mérieux Laboratory, Chinese Academy of Medical Sciences & Peking Union Medical College National Institute of Pathogen Biology, Beijing, P. R. China.

^3^Key Laboratory of Pathogen Infection Prevention and Control (Ministry of Education), State Key Laboratory of Respiratory Health and Multimorbidity, National Institute of Pathogen Biology, Chinese Academy of Medical Sciences & Peking Union Medical College, Beijing, P. R. China.

^4^Key Laboratory of Respiratory Disease Pathogenomics, Chinese Academy of Medical Sciences & Peking Union Medical College, Beijing, P. R. China.

^#^ Equal contribution as co-first authors

^*^ Equal contribution as co-senior authors

**Table S1.** **Reagents used in this study.**

| **Reagent** | **Source** | **Identifier** |
| --- | --- | --- |
| **Recombinant proteins** |  |  |
| Monkeypox virus A29L protein | ACROBiosystems | A2L-M52H3 |
| Monkeypox virus A35R protein | ACROBiosystems | A3R-M52H3 |
| Monkeypox virus E8L protein | ACROBiosystems | E8L-M52H3 |
| Monkeypox virusH3L protein | ACROBiosystems | H3L-M52H1 |
| Monkeypox virus B6R protein | Sino Biological | 40902-V08H |
| Monkeypox virus M1R protein | Sino Biological | 40904-V07H |
| **Antibodies** |  |  |
| Horseradish peroxidase-conjugated goat anti-human Fc5μ fragment-specific polyclonal IgM | Jackson ImmunoResearch | 109-035-043 |
| Horseradish peroxidase-conjugated goat rabbit anti-human α chain-specific polyclonal IgA | Sigma Aldrich | A0295 |
| Horseradish peroxidase-conjugated goat anti-human Fc-specific polyclonal IgG | Sigma Aldrich | A1070 |
| **Critical commercial assays** |  |  |
| Viral DNA and RNA Extraction Kit | Xi’an Tianlong Science & Technology Co., Ltd. | T324H |
| Real-time PCR Detection Kit for Monkeypox Virus | Beijing Applied Biological Technologies Co., Ltd. | D2361YH |
| Pierce™ BCA Protein Assay Kit | Thermo Fisher Scientific | 23227 |
| Human Immunodeficiency Virus Type I (HIV-1) Nucleic Acid Detection Kit | WANTAI Biopharm | IPQ20230903 |
|  |  |  |

**Table S2. Demographics, HIV status and self-reported symptoms of the 10 PLWH with seroconversion.**

| Patient  No. | Year of  birth | Sex | CD4 cell  count  (cells/mm^3^) | HIV  viral load (copies/mL) | Fever  (°C) | Skin lesions | Site of  lesions | No. of lesions | Headache | Sore throat | Swollen lymph nodes | Muscle pain | Diarrhoea | Vomiting | See a doctor | Self-medication |
| --- | --- | --- | --- | --- | --- | --- | --- | --- | --- | --- | --- | --- | --- | --- | --- | --- |
| P1 | 1977 | Male | 882 | 1944 | No | No | / | / | No | No | No | No | No | No | No | No |
| P2 | 1983 | Male | 651 | 5589 | No | Yes | Upper limb | 5–10 | No | No | No | No | Yes | No | No | No |
| P3 | 1988 | Male | 938 | 120 | No | No | / | / | No | No | No | No | No | No | No | No |
| P4 | 1969 | Male | 611 | 30 | No | No | / | / | No | No | No | No | No | No | No | No |
| P5 | 1994 | Male | 877 | ND | 38°C | No | / | / | No | No | No | No | Yes | Yes | Yes | Yes |
| P6 | 1980 | Male | 855 | 6884 | No | No | / | / | No | No | No | No | No | No | No | No |
| P7 | 1971 | Male | 1026 | 34436 | 37.5°C | No | / | / | No | No | No | No | Yes | No | Yes | Yes |
| P8 | 1989 | Male | 645 | 702 | No | No | / | / | No | No | No | No | No | No | No | No |
| P9 | 1983 | Male | 775 | ND | No | No | / | / | No | No | No | No | Yes | No | No | No |
| P10 | 1986 | Male | 728 | 11695 | No | No | / | / | No | No | No | No | No | No | No | No |

PLWH=people living with HIV

ND=Not detectable

**Figure S1. Kinetics of MPX- IgM titers in 10 participants**

Kinetics of the MPXV-IgM titers of 10 participants from baseline to follow-up. The dotted lines indicate the detection limits of the assays.
